# Amplification of multiple genomic loci from single cells isolated by laser micro-dissection of tissues

**DOI:** 10.1186/1472-6750-8-17

**Published:** 2008-02-20

**Authors:** Dan Frumkin, Adam Wasserstrom, Shalev Itzkovitz, Alon Harmelin, Gideon Rechavi, Ehud Shapiro

**Affiliations:** 1 Department of Biological Chemistry, Weizmann Institute of Science, Rehovot, Israel; 2 Department of Computer Science and Applied Mathematics, Weizmann Institute of Science, Rehovot, Israel; 3 Department of Veterinary Resources, Weizmann Institute of Science, Rehovot, Israel; 4 Sheba Cancer Research Center and the Institute of Hematology, The Chaim Sheba Medical Center, Tel Hashomer and the Sackler School of Medicine, Tel Aviv University, Israel

## Abstract

**Background:**

Whole genome amplification (WGA) and laser assisted micro-dissection represent two recently developed technologies that can greatly advance biological and medical research. WGA allows the analysis of multiple genomic loci from a single genome and has been performed on single cells from cell suspensions and from enzymatically-digested tissues. Laser micro-dissection makes it possible to isolate specific single cells from heterogeneous tissues.

**Results:**

Here we applied for the first time WGA on laser micro-dissected single cells from stained tissue sections, and developed a protocol for sequentially performing the two procedures. The combined procedure allows correlating the cell's genome with its natural morphology and precise anatomical position. From each cell we amplified 122 genomic and mitochondrial loci. In cells obtained from fresh tissue sections, 64.5% of alleles successfully amplified to ~700000 copies each, and mitochondrial DNA was amplified successfully in all cells. Multiplex PCR amplification and analysis of cells from pre-stored sections yielded significantly poorer results. Sequencing and capillary electrophoresis of WGA products allowed detection of slippage mutations in microsatellites (MS), and point mutations in P53.

**Conclusion:**

Comprehensive genomic analysis of single cells from stained tissue sections opens new research opportunities for cell lineage and depth analyses, genome-wide mutation surveys, and other single cell assays.

## Background

Recent years have seen the birth of the single cell analysis era. With the development of technology, many research procedures and assays that were previously performed only on populations of cells have recently been applied for the study of single cells (reviewed in [1]). These include PCR [2], RT-PCR [3], comparative genomic hybridization [4], and two-dimensional electrophoresis [5].

The development of WGA methods (reviewed in [6,7]) has recently made it possible to analyze multiple genomic loci from single cells. Early methods were based on PCR and included degenerate oligonucleotide-primed (DOP) PCR [8], primer extension pre-amplification (PEP) [9], and ligation-mediated PCR [10]. PCR-based WGA techniques were used to amplify genomes of single cells such as blastomeres in pre-implantation genetic diagnosis (reviewed in [11,12]), lymphocytes [13], hepatocytes [14], sperm [9], oocytes [15], bone marrow cells [16,17], and even single chromosomes [18,19].

Based on a combination of PEP and DOP, a commercial kit for single cell WGA was developed and used to amplify single human [20] and mouse cells (Wasserstrom, A. *et al*, submitted).

Recently, multiple displacement amplification using Φ29 DNA polymerase [21] was developed as an isothermal, non PCR-based method for WGA. Multiple displacement amplification results in better genomic coverage and in less biased-amplification than earlier PCR-based methods [6]. In addition, due to the low error rate of Φ29 [21], it results in less artificial mutations than PCR-based methods [6,7], and this may be especially important for single cell WGA, since erroneous copying of the single template molecule in the first stages of the reaction might result in a false genotype. Multiple displacement amplification was performed on several types of single cells, including bacteria [22], fungal pores [23,24], human blastomeres [25,26], lymphocytes [25,27-29], buccal [30], and sperm cells [31].

All of the aforementioned single cell WGA reactions were performed either on non adhering cells (e.g. sperm, blood cells) or on cells that were obtained from tissues by mechanical disruption and/or enzymatic digestion of the tissue and re-suspension. While valuable information can be obtained via this approach, the disruption or digestion of tissues carries a disadvantage since it destroys the natural architecture and thus results in loss of information regarding the morphology and precise anatomical position of the isolated cells. This disadvantage is compounded by the fact that the structure of animal tissues is inherently complex, consisting of many different cell types in close proximity [32], and therefore biological research of tissue micro-environments requires a more subtle approach to cell isolation.

In order to preserve data regarding morphology and position, cells can be cut from stained tissue sections by micro-dissection, either manually or by laser assisted micro-dissection. Manual micro-dissections under an inverse microscope were used in conjunction with PCR [2] and with a PCR-based WGA method [33] to study T-cell receptor and P53 gene sequences in single human cells.

The use of laser for micro-dissection has many advantages over manual micro-manipulation and is now considered the method of choice for obtaining pure cell populations or single cells from mixed tissues [32]. Laser micro-dissection utilizes a computer-aided robot in conjunction with a microscope and a laser machine, enabling easy isolation of any desired cell with very high precision and reproducibility. Moreover, the recent incorporation of pressure catapulting in laser micro-dissection [34] allows for contact-free direct transfer of cells, thus reducing the risk for contamination.

Laser micro-dissection has been used in conjunction with a variety of downstream molecular techniques for characterization of the genome, transcriptome, and proteome of pure populations of cells in normal and pathological conditions (reviewed in [32]). The combination of laser micro-dissection followed by WGA has recently emerged as a powerful tool for large scale genomic analyses of pure populations of cells, and it was used to characterize genetic alterations in pathologic conditions such as chronic pancreatitis [35], motor neuron disease [36], and various pre-malignant [37], and malignant tumors [37-40]. In these studies, the number of micro-dissected cells used as starting material for WGA ranged from 50 to 1000s. In one study, accurate genotyping was demonstrated from as little as 100 cells [39].

The high precision of laser micro-dissecton allows for isolation of single cells and even sub-cellular components, such as nuclei, nucleus free cytoplasm, and chromosomes [32]. Laser micro-dissection followed by RT-PCR was recently used to examine expression of specific genes in single cells from frozen human brain [41-43] and muscle [44] tissues. Laser micro-dissection followed by PCR was also used on single cells from formalin-fixed, paraffin-embedded (FFPE) tissues for detection of latent viral infection in human Trigeminal ganglia [45].

Here we utilized laser micro-dissection coupled to pressure catapulting followed by multiple displacement amplification for analysis of multiple genomic loci from single cells obtained from frozen mouse tissue sections. For each cell, we amplified over 100 genomic loci, including microsatellites (MS), gene exons, and mitochondrial DNA, and analyzed the amplified products by sequencing, capillary and gel electrophoresis.

## Results

### Cell amplification procedure

**Figure 1:**
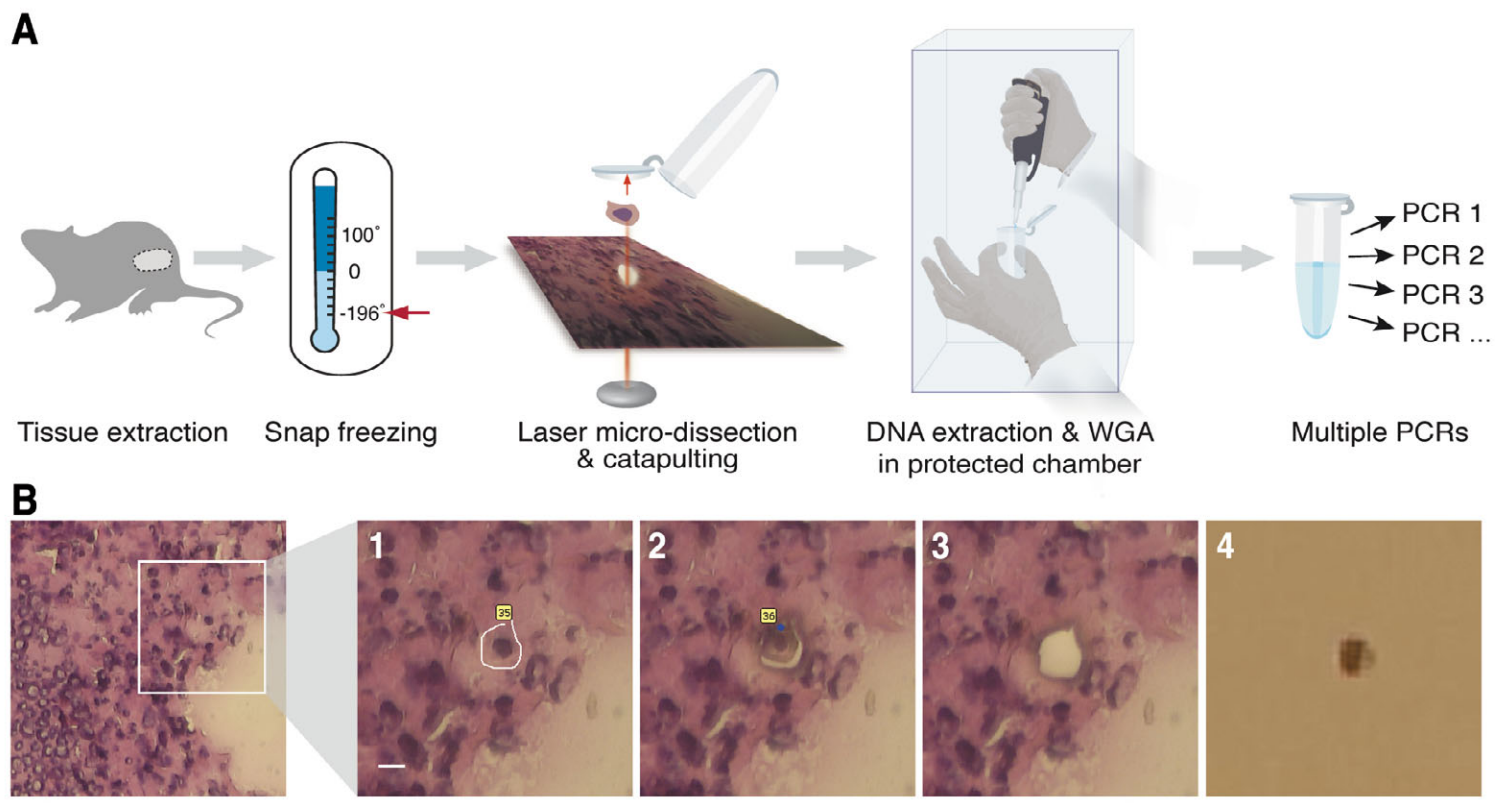
Single cell genome amplification procedure. (A) Tissues of interest are excised and snap frozen in liquid nitrogen. After sectioning, staining, and mounting on a polyethylene membrane coated slide, a cell of interest is laser micro-dissected and catapulted into an adhesive cap of a micro-centrifuge tube. The cell is then subject to DNA extraction and WGA in a protected chamber, minimizing the chance for contamination. Aliquots of the WGA products are amplified by multiple PCRs with specific primers for analysis of multiple genomic loci. (B) Serial photographs taken during laser micro-dissection and catapulting of a single cell. The left panel shows a stained tissue section under low magnification. A portion of the tissue section is viewed under high magnification before (1) and after (2) micro-dissection, and after catapulting (3) of the single cell (bar = 6 μm). Inspection of the adhesive cap under low magnification (4) reveals a catapulted single cell.

The procedure for amplifying genomes of laser micro-dissected single cells from frozen tissue sections is outlined in Figure 1A. (full protocols are presented in the materials and methods section). Tissue freezing, sectioning, and staining are performed using standard protocols. In order to minimize the risk of laser-induced damage to DNA, thin (6 μm) sections are used, and micro-dissection is performed using the lowest possible energy level (determined empirically for each section, see materials and methods). Laser ablation paths are typically less than 1 μm in thickness, allowing for isolation of intact cell nuclei with contiguous cytoplasm (Figure 1B). DNA extraction and WGA are performed using the GenomiPhi DNA amplification kit reagents and protocol, with modifications. In order to reduce the risk of contamination from extraneous DNA sources, which is a major concern in single-cell procedures, stringent precautions are employed. These include prior treatment of work surfaces with DNA-destroying agents, use of dedicated pipettes and other instruments, performing DNA extraction and WGA in a restricted chamber with a UV lamp, and the use of a blank control from each tissue section in subsequent WGA and PCR reactions. Aliquots of amplified products can be used directly (without purification) as DNA templates in PCRs for analyses of specific genomic loci.

We used this procedure to amplify the genomes of 37 single cells from sections of frozen mouse tumor and normal lung tissues, which were micro-dissected and catapulted onto micro-tube caps. A total of 41 DNA extractions and WGA reactions were initially performed. However, since catapulting was not always efficient, and visual scanning of the caps could not always detect the presence of cells with certainty, the WGA products were subject to a preliminary multiplex PCR assay with primers for 4 MS loci. Out of the 41 samples, 4 showed no amplification in any locus in the preliminary assay and were thus considered to contain no cell and were discarded. The remaining 37 samples were processed further by multiple PCRs followed by capillary electrophoresis and signal analysis. Out of the 37 cells, 20 were obtained from fresh tissue sections (i.e. micro-dissection was performed immediately following preparation of the tissue section), and 17 were obtained from pre-stored tissue sections (i.e. micro-dissection was performed on sections that were prepared earlier and stored in -80°C for up to several weeks). For each single cell sample, as well as for the tail clipping DNA, 122 genomic loci were amplified (following WGA). Most analyzed loci were 100–500 bp long. In order to determine if longer fragments could be analyzed, a 2.5 kbp fragment (P53long) was also amplified.

### DNA size range and yield

**Figure 2:**
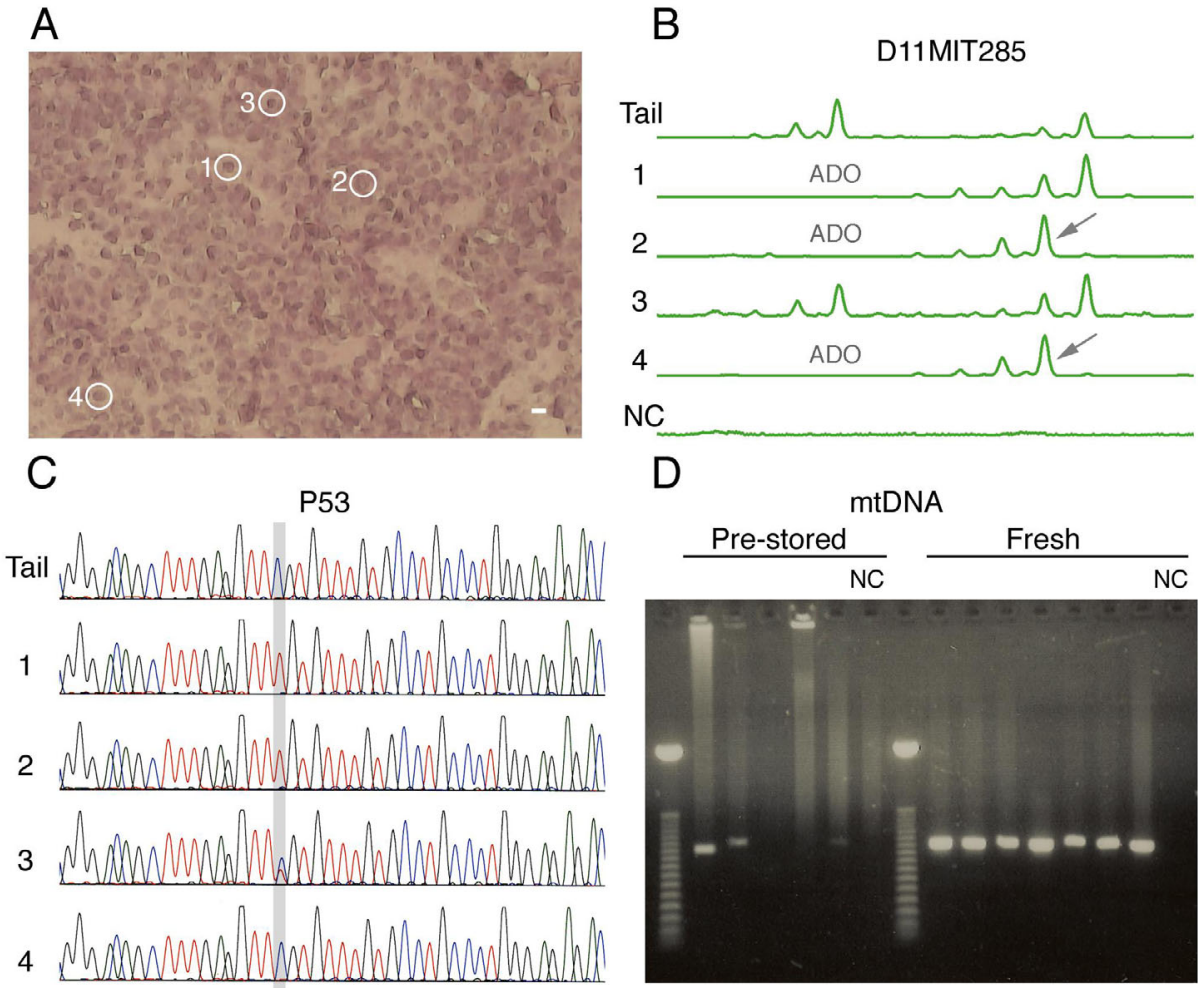
Amplification of multiple genomic loci from single cells. (A) Tissue section with cells 1–4 marked by white circles (bar = 6 μm). (B) MS locus ABI20 was analyzed by PCR amplification and capillary electrophoresis. Both paternal and maternal alleles are visible in the tail clipping sample (Tail) and in cell 3. Allelic dropout (ADO) can be seen in the short allele of cells 1, 2, and 4. Slippage mutations (arrows) can be seen in the long allele of cells 2 and 4. The negative control (NC) sample shows no amplification. (C) Sequencing of exon 8 from the P53 gene. A point mutation (C => T) can be seen in the 18^th ^nucleotide from the left (highlighted grey) in cells 1 and 2. In cell 3, both the normal and mutated alleles were amplified, and in cell 4 only a normal allele is visible. (D) Mitochondrial ND3 gene locus was amplified by PCR and run on an agarose gel. Amplification is successful for all cells from a fresh section, but not for all cells from a pre-stored section.

The size-range of WGA products was assessed by agarose gel electrophoresis. Amplified products produced a pattern similar to un-amplified tail clipping DNA, namely a smear ranging in size from ~500 bp upward to several kbp (Additional File 1). As expected, electrophoresis of negative control samples (i.e. no input DNA) also produced a similar pattern. However, negative control samples did not produce signals in subsequent specific PCRs (see example in Figure 2B), confirming that the observed DNA was non-specific.

Quantification of total DNA yield was performed for 8 WGA products, and yields ranged from 5.5–17 μg (mean 9.7 μg ± 4.2 μg). However, since non-specific DNA is present in the negative control samples, it is likely that some non-specific DNA is also present in WGA products of cells, and therefore specific amplification cannot be accurately inferred from total yield. Therefore, in order to estimate specific yield and fold-amplification, we performed quantitative real time PCRs (see below).

### Genomic coverage

For each of the 37 single cell WGA products, as well as for the tail clipping DNA, 122 nuclear genomic loci (120 MS loci, and two gene segments – P53 and SRY) were amplified and analyzed. In addition, P53long and the mitochondrial ND3 locus were also amplified and analyzed (discussed below). Of the 122 loci, 32 were amplified in separate (singleplex) PCR reactions, while the remaining 90 loci were amplified by 24 multiplex PCRs, each containing primers for 3–4 MS loci. Information on each locus, including primer names, sequences (where available), basic repeat units (for MS loci), and genomic positions is presented in Table 1. All PCRs were performed with fluorescent forward primers, and capillary electrophoresis signals of PCR products were analyzed and compared to the signal from the tail clipping DNA.

**Table 1:** Loci information and amplification statistics

	**Name**	**Repeat**	**Chr**	**Forward primer**	**Reverse primer**	**Amp/L (%)**	**Amp/A (%)**	**ADO (%)**
Loci amplified by multiplex PCRs (1–90)								

1	D16MIT189	AC	16	N/A	N/A	70.0	45.0	71.4
2	D9MIT201	TG	9	N/A	N/A	75.0	57.5	46.7
3	D12MIT182	TG	12	N/A	N/A	75.0	42.5	86.7
4	D4MIT203	TG	4	N/A	N/A	65.0	35.0	92.3
5	D17MIT180	TG	17	N/A	N/A	80.0	45.0	87.5
6	D1MIT316	AC	1	N/A	N/A	50.0	30.0	80.0
7	D1MIT206	TG	1	N/A	N/A	65.0	45.0	61.5
8	D9MIT250	AC	9	N/A	N/A	80.0	52.5	68.7
9	D4MIT17	TG	4	N/A	N/A	80.0	57.5	56.2
10	D5MIT425	AC	5	N/A	N/A	55.0	30.0	90.9
11	D18MIT222	TC	18	N/A	N/A	85.0	85.0	0
12	D4MIT18	TG	4	N/A	N/A	70.0	45.0	71.4
13	D7MIT259	AC	7	N/A	N/A	75.0	42.5	86.6
14	D10MIT213	TG	10	N/A	N/A	75.0	37.5	100
15	D9MIT198	TG	9	N/A	N/A	70.0	47.5	64.3
16	D10MIT95	AC	10	N/A	N/A	65.0	35.0	92.3
17	D14MIT170	TG	14	N/A	N/A	60.0		
18	D19MIT88	GA	19	N/A	N/A	85.0	50.0	82.3
19	D15MIT44	AC	15	N/A	N/A	25.0	15.0	80.0
20	D5MIT146	TG	5	N/A	N/A	80.0	52.5	68.7
21	D8MIT45	AC	8	N/A	N/A	85.0	50.0	82.3
22	D15MIT159	AC	15	N/A	N/A	70.0	45.0	71.4
23	D19MIT33	TC	19	N/A	N/A	80.0	62.5	43.7
24	D6MIT36	TG	6	N/A	N/A	55.0	40.0	54.5
25	D10MIT233	AC	10	N/A	N/A	80.0	47.5	81.2
26	D1MIT132	GA	1	N/A	N/A	55.0	32.5	81.8
27	D19MIT26	TG	19	N/A	N/A	60.0	35.0	83.3
28	D6MIT284	AC	6	N/A	N/A	65.0	42.5	69.2
29	D8MIT292	AC	8	N/A	N/A	70.0	42.5	78.5
30	D8MIT120	AC	8	N/A	N/A	70.0	45.0	71.4
31	D2MIT285	TG	2	N/A	N/A	55.0	40.0	54.5
32	D11MIT285	AC	11	N/A	N/A	70.0	50.0	57.1
33	D11MIT143	AC	11	N/A	N/A	80.0	50.0	75.0
34	D1MIT102	TG	1	N/A	N/A	75.0	50.0	66.6
35	D11MIT86	TG	11	N/A	N/A	80.0	42.5	93.7
36	D2MIT242	TG	2	N/A	N/A	65.0	35.0	92.3
37	D14MIT126	AC	14	N/A	N/A	90.0	57.5	72.2
38	D14MIT174	TG	14	N/A	N/A	75.0		
39	D2MIT208	AC	2	N/A	N/A	75.0		
40	D5MIT98	AC	5	N/A	N/A	25.0		
41	D18MIT194	TG	18	N/A	N/A	85.0	55.0	70.5
42	D13MIT19	GA	13	N/A	N/A	80.0	45.0	87.5
43	D17MIT122	AC	17	N/A	N/A	70.0	42.5	78.5
44	D12MIT91	AC	12	N/A	N/A	65.0	40.0	76.9
45	D5MIT10	AC	5	N/A	N/A	70.0		
46	D3MIT203	TG	3	N/A	N/A	90.0	70.0	44.4
47	D4MIT209	TG	4	N/A	N/A	60.0	32.5	91.6
48	D15MIT161	TG	15	N/A	N/A	85.0	47.5	88.2
49	D1MIT64	AC	1	N/A	N/A	70.0	42.5	78.5
50	D14MIT60	AC	14	N/A	N/A	70.0	57.5	35.7
51	D12MIT59	AC	12	N/A	N/A	70.0	50.0	57.1
52	D1MIT440	TG	1	N/A	N/A	45.0	32.5	55.5
53	D9MIT336	AC	9	N/A	N/A	70.6	41.2	83.3
54	D13MIT16	TG	13	N/A	N/A	65.0	25.0	69.2
55	D2MIT1	AC	2	N/A	N/A	85.0	55.0	70.5
56	D4MIT348	AC	4	N/A	N/A	80.0	45.0	87.5
57	H610	G	14	ctacagtagtagcatgagaggtggtg	caaaagaatttctccttttacattgg	85.0		
58	Barvaz	GA	1	tggctgcacaaacaagataggag	cgaaacgtgctgtgtccattttg	30.0	15.0	100
59	L2924	AAAGGG	12	ggtgggtctctgtgggtttgag	tcatctccattagcacctgagcac	40.0		
60	L0382	AG	5	catcgtggaaactgacccttcc	tgtgaaggcaccaaaattgagtttc	80.0	57.5	56.2
61	D2MIT66	AC	2	gttgcacaggcaatcaacc	atctatcactggggctgtgc	40.0		
62	D2MIT411	AC	2	acactcacaactacgagataaagcc	aggtcattagggctgtcttcc	52.9	26.5	100
63	D1MIT1001	AC	1	ttgtgtgtagtacagtgttggtgg	tggttcctgacatcaatctcc	85.0		
64	D2MIT100	AC	2	gtgttcctaaggttgtattttggc	gaaatttgacaattgctaggtgc	80.0	50.0	75.0
65	D1MIT426	AC	1	ctgccatccactacttggtg	caaatgatacagtggaaacccc	85.0	60.0	58.8
66	L2621	AC	12	atgaaaagatgcaaattccagcac	aggctgccatacactcctccag	80.0	70.0	25.0
67	L3464	AAG	16	ttcagtctcctcccatctgtgc	cgatgtgttgtgcattggttcc	70.0	40.0	85.7
68	L2454	AGTC	17	ttccccacatcgctgtaaatgg	tggcctgagacaaaagcctagc	84.6	61.5	54.5
69	M4	AAG	6	acggcgtgccttttcattttac	cttgtcccttgctgctcatctg	65.0	42.5	69.2
70	L2462	AAAGC	3	ccagagatacatagtgagaccatgacg	ctgatggtcctgctggcttttag	65.0	55.0	30.7
71	L6231	AGC	5	actccccacagaggtcaccaag	gctggctctcctgtagacattgg	65.0	47.5	53.8
72	L1053	AAAG	7	aggcctatctttgccgcagac	gcctggcattgtatctcaggttc	35.0	27.5	42.8
73	L2492	AAAGG	5	acccacatagaggcagggtgag	tcacagactgagttgaaggagaagg	40.0	25.0	75.0
74	M16	AAGC	10	ttcaggtagatacatcagacctgtgg	aagtcttgggggaacagtcgag	75.0	45.0	80.0
75	M19	TCTCT	10	tgtgcagggaagactggatctg	tgatcatctcaagtgttttgtcacg	75.0	45.0	80.0
76	H341	G	12	gcctaggacataaggatggtagattg	taggttgatatgtgagtgcaaagaaag	25.0		
77	L1463	CCTCTTCTT	16	catcaccccagctctttgaatc	tcccagaaatatgttgaacttcagtc	40.0	22.5	87.5
78	M2	AC	5	aggccacacctgagcttttagc	tcttcccaatcaccgattcacc	70.0		
79	M7	AAG	6	tccagccttcagtaggcacagg	ggacaactaccacaaaattccaagg	60.0	37.5	75.0
80	M11	TTTC	11	aaactttatcaggaggaaagtgaaagc	ggccacatcacttttgaagctg	35.0	20.0	85.7
81	M6	AGA	1	ggacaaaccaatgtgttcttgtgtg	tgagcagcatctctggagaacag	80.0	40.0	100
82	M8	TCC	X	aagttgcccagaggggaatgtc	ttcatggaaataaacatgcttctgg	40.0	40.0	
83	M18	AAGCA	3	agaccaggcaccaccagtcaag	cgtaaagaacgcagataaagcttgc	65.0		
84	M15	CTAT	18	acttggaggaggacggtgagag	tttacttagtgctcagcttggaagg	65.0	45.0	61.5
85	L2941	AG	1	gtaggcctgcaaagcaggagtg	ggtctgggctagggtgggaag	75.0	47.5	73.3
86	D1MIT495	TC...AC	1	ccaccttgctccaaaagaaa	tctgagaggctgccacaata	75.0	47.5	73.3
87	L7262	AAC	2	ctgagttggcaggcaaaatgtg	ttgcctctcaagcctttgtgtc	71.4	46.4	70.0
88	L4163	GCCTCCT	2	tggctggactgagattccacag	caaaccctttagcagagcatgg	70.0	47.5	64.3
89	M13	CTTT	14	gggcataaattgtttgtcgcttg	gtgtgactgctcgcttcccatc	60.0	35.0	83.3
90	M17	AGAA	X	tctcatggatgaacctataaacaaagg	aattgaaaagtgtgagcccatgc	35.0	35.0	

Loci amplified by singleplex PCRs (91–124)								

91	IDT5	TC	13	gagccaactctatgggctgagg	catagcaaccccatccttcctg	80.0	60.0	50.0
92	IDT7	AG	13	gcccctgaatcttgaactggtg	ccccaaaagtagccaacagtgg	80.0	55.0	62.5
93	IDT8	GA	6	catacagtgccccctccctaag	agctttcctgaggggcattctc	90.0	70.0	44.4
94	IDT12	AG	4	gaatagcatcaccgcactgcac	agaggtccgttgcatctgttgg	80.0	42.5	93.7
95	IDT13	AC	2	ggagggttttaaatagggaatgtgaag	tgcaaagtgcccttctttgacc	90.0	60.0	66.6
96	IDT14	TC	14	cgaactctttgcctcctgtatttcc	cagaatctggacaccacaacattacac	95.0	62.5	68.4
97	LX1	A	X	ccgaggatctttcctcgtttattg	ttcatgctgtcccagaccagtg	80.0	80.0	
98	LX6	G	X	tggcagccatagttcattcagg	agtaggggaaatggcagggttg	80.0	80.0	
99	LX7	C	X	catgtgaaagtggtgtcaacttgg	cagtatttggtggcctttcatcg	50.0	50.0	
100	LX10	GA	X	cccgacttcctgcttcttttcc	cattccttcatcccctccttcc	80.0	80.0	
101	LX11	A	X	cctttctgcttggggttctgtg	ggaaaaggaagtgcagggagag	75.0	75.0	
102	LX33	GA	X	tgaactctggtcaatcatctcacag	ccccaaagcatttacacatataggg	75.0	75.0	
103	LX39	GT	X	ttgtcccaagagttccacaagg	accagtatggccaaaggagcag	80.0	80.0	
104	LX46	A	X	ggaaggggaaacaaccaaaatg	cccacttgtagaacagtttgccttc	70.0	70.0	
105	LX12	T	X	ttctagtccatccagcccttcc	ggggcgtgctgtaccttaattg	75.0	75.0	
106	LX35	TC	X	atgagcaggaggaggagtgctg	aagagcgagaaatgacgcaagg	80.0	80.0	
107	LX40	AC	X	aggaccccatctcttggtttgg	gccagccttgaggaatacaacg	40.0	40.0	
108	LX27	A	X	caactcagttcccccatgacac	tccaaaaaccaggcaattctcc	80.0	80.0	
109	LX42	GT	X	tcatagaccccaaactggctgtc	tggagcagcctagtggaattgtc	80.0	80.0	
110	LX43	CA	X	tcttttgtggatgccagagtcaag	tggatatgggcattgaatcttcg	75.0	75.0	
111	LX18	T	X	gaagagcctcagctgcaaggac	cgccaatcaaccccatttttag	75.0	75.0	
112	LX47	GT	X	gagccaacaaggtccctgaaac	ggggagcatttgctgaattacc	75.0	75.0	
113	LX17	G	X	caccatcagcctttcccaagac	cctctctggctttgctttctgg	65.0	65.0	
114	LX20	T	X	ggcatcctcgctattccatgag	caaatgctgtggaattcaccaatg	80.0	80.0	
115	LX25	GA	X	tattgcctgtggaagggattgg	ggcaatgccatttggctcttag	35.0	35.0	
116	LX28	TC	X	tggattccgatattcaacaatacatcc	ctgagcactctgcgagcaaaac	50.0	50.0	
117	LX34	CT	X	cagcaaaaacaggtggctgtg	aatgcagggctcaggaaatgag	35.0	35.0	
118	LX32	AG	X	atgttcaatgcatcccctctcc	tgatggggactcagagttttcg	45.0	45.0	
119	LX31	GA	X	tgatgccatccaaaatcatcatc	gccaggtaggaagatggtcagtc	45.0	45.0	
120	LX24	A	X	cggggacattccacgttagttc	gcttatggtggattccctgtgc	40.0	40.0	
121	SRY		Y	gtgagaggcacaagttggc	ctctgtgtaggatcttcaatc	85.0	85.0	
122	P53short		11	Ttcttactgccttgtgctggtc	aagaggtgactttggggtgaag	85.0		
123	P53long		11	Acacctgatcgttactcggcttgtc	ttcactacaaaggctgagctgg	64.7		
124	ND3		Mt	acgtctccatttattgatgagg	gaggttgaagaaggtagatggc	100.0		

Amp/L – amplification per locus

Amp/A – amplification per allele. Calculated for heterozygous and hemizygous loci.

ADO – allele drop out. Calculated for heterozygous loci

N/A – Not available

In order to assess genomic coverage, we defined 4 parameters. Amplification per locus (Amp/L) was defined as amplification of at least one allele in a locus, and was calculated for all heterozygous, homozygous, and hemizygous loci (on chromosomes X and Y). Amplification failure was defined as 1-Amp/L and was a measure of the fraction of genomic loci that completely failed to amplify. Amplification per allele (Amp/A) was defined as amplification of individual alleles in a locus, and was calculated in heterozygous and hemizygous loci, but not in homozygous loci, where it is not possible to distinguish between amplification of one allele from amplification of both alleles (note that in hemizygous loci, Amp/L and Amp/A necessarily have equal values). Allele drop out (ADO) was defined as failure of amplification of one out of two alleles in a heterozygote locus. Amplification and drop out rates for each genomic locus, calculated from samples obtained from fresh tissue sections, are presented fully in Table 1, and summarized in Table 2. Loci amplified by singleplex PCR performed better than loci that were amplified by multiplex PCR, as demonstrated by significantly higher rates of Amp/L (70.3% ± 17.4% vs. 66.9% ± 15.9%; p = 0.043), Amp/A (64.5% ± 16.1% vs 44.2% ± 11.8%; p < 10^-7^), and lower rates of ADO (64.2% ± 17.2% vs. 71.7% ± 18.3%; p = 0.055). Amplification and drop out rates across all loci were also calculated separately for each of the 37 single cell samples and are summarized in Table 3 (full results are presented in Additional File 2). Amplification in cells obtained from fresh tissue sections was better than in cells that were obtained from pre-stored tissue sections, as demonstrated by significantly higher rates of Amp/L, Amp/A, and lower rates of ADO in both singleplex and multiplex PCRs (Table 3).

**Table 2:** Mean amplification and dropout rates for genomic loci

	Amp/L (%)	Amp/A (%)	ADO (%)
Singleplex	70.3 ± 17.4	64.5 ± 16.1	64.2 ± 17.2
Multiplex	66.9 ± 15.9	44.2 ± 11.8	71.7 ± 18.3
P value	0.043	8.3×10^-8^	0.055

Each Amp/L, Amp/A, and ADO value represents mean ± standard deviation of the corresponding distribution of values presented in Table 1 (excluding P53long and ND3).

P values represent Kolmogorov-Smirnov goodness-of-fit hypothesis test scores for comparisons between the respective singleplex and multiplex distributions.

**Table 3:** Mean amplification and dropout rates for cells

	Singleplex			Multiplex		
	Amp/L	Amp/A	ADO	Amp/L	Amp/A	ADO
Fresh	70.3 ± 15.5	63.5 ± 16.3	64.2 ± 27.6	66.9 ± 12.9	45.4 ± 10.5	71.9 ± 13.9
Pre-stored	36.5 ± 15.5	32.9 ± 14.2	75.5 ± 28.4	40.4 ± 12.6	24.7 ± 7.5	85.3 ± 3.8
P score	2.1×10^-5^	3.6×10^-5^	0.48	2.1×10^-5^	2.1× ^-5^	3.7×10^-4^

Each result represents mean percentage ± standard deviation of the corresponding distribution that is presented in Additional file 2. For fresh cells, mean amplification values differ slightly from corresponding rates in table 1, because absence of uncounted samples (due to positive signals in the corresponding negative controls) tend to affect the average slightly differently in horizontal vs. vertical calculation (i.e. when calculated per cell vs. per locus).

P scores represent Kolmogorov-Smirnov goodness-of-fit hypothesis test scores for comparisons between the respective distributions.

Cells from fresh tissue sections that were amplified by singleplex PCR performed best, achieving Amp/L rates between 37.5 – 96.8% (mean 70.3% ± 15.5%), Amp/A rates between 29.7% – 91.8% (mean 63.5% ± 16.3%), and a mean ADO rate of 64.2%. In both fresh and pre-stored sections, ADO appeared to occur randomly, with different cells displaying dropout of the maternal or paternal allele (see example of ADO in Figure 2B). However, longer alleles (i.e. with a larger number of repeats – either paternal or maternal) had significantly higher dropout rates in comparison to their corresponding shorter alleles: out of a total of 801 ADO events, 445 (55.6%) occurred in the long alleles and 356 (44.4%) occurred in the short alleles (P = 0.0007; calculated for all 37 cells). This bias towards dropout of the longer allele is most likely the result of preferential amplification of the shorter allele during PCR [46].

### Correlation between amplification rate and physical location of loci

We suspected that some of the observed amplification failure and ADO was a result of partial or complete loss of chromosomes as a result of truncation of nuclei during tissue sectioning. To test this possibility, we calculated the concordance of amplification status for pairs of loci on the same chromosome and compared it to the concordance of amplification status for pairs of loci on different chromosomes (in this analysis maternal and paternal homologues were considered different chromosomes).

The concordance for loci within a chromosome was 60.9% (7305 out of the 11988 same-chromosome pairs that were analyzed were both amplified or both not-amplified), while the concordance for loci on different chromosomes was 56.3% (52527/93166 pairs analyzed). In order to determine whether the higher concordance in amplification status that was observed for loci on the same chromosome was statistically significant, we performed a random permutation test. We performed 1000 permutations of all pairs of loci, and for each permutation we randomly assigned "same chromosome" and "different chromosomes" groups of pairs and calculated their respective concordance values. In all 1000 permutations, the difference in concordance rates between the groups was lower than the observed difference in the real groups, and therefore we conclude that pairs of loci on the same chromosome have significantly higher rates of concordance in amplification status than pairs of loci on different chromosomes (p < 0.001). Further, we sought to determine whether there is a correlation between concordance in amplification status and physical distance between loci on the same chromosome. For this purpose we analyzed the amplification status of 24 MS loci with known physical locations on the X chromosome. We created a matrix of physical Euclidean distances between the loci along the chromosome, and a second matrix of concordance of amplification between these same loci. The correlation of these matrices was -0.08, indicating that smaller physical distances were associated with higher amplification concordance, although this result was not significant relative to a permutation test (performed by permuting one of the matrices 1000 times and re-computing the correlation coefficient, p = 0.13). Together, these results support the possibility that truncation of nuclei during tissue sectioning resulted in loss of genetic information.

### Specific yield and fold amplification

For calculating specific DNA yield and fold-amplification, quantitative real-time PCR was performed on 8 cells from fresh tissue sections, using primers for 4 genomic MS loci and the mitochondrial ND3 locus. The specific genomic loci were chosen because they were single copy, hemizygous loci, from chromosomes X and Y, that amplified successfully in all 8 cells in regular PCRs. Hemizygosity was important because fold-amplification can be measured accurately in hemizygous loci, where all amplification necessarily stems from the single genomic copy, in contrast to heterozygous or homozygous loci, where amplification can stem from either two or just one of the genomic copies (due to ADO). Quantitative real-time PCR results are presented in Table 4. Single copy genomic loci were amplified to 3.8×10^5 ^– 1.2×10^6 ^copies (mean 6.9×10^5 ^± 3.4×10^5^), and specific DNA represented 18.4–60.7% (mean 44.2% ± 12.1%) of total DNA (specific + non-specific). However, this calculation of specific DNA percentage and yield represented only genomic loci that amplified successfully (loci used for quantitative real-time PCR were chosen for this purpose because of their successful amplification in regular PCRs). Therefore, in order to calculate the corresponding un-biased figure for all loci, the figure for specific DNA percentage was multiplied by the mean Amp/A for all loci (63.5%), yielding a mean corrected specific DNA percentage of 28.0%.

**Table 4:** Percentage and fold-amplification of specific DNA determined by real-time PCR

	Real-time PCR concentration (ng/μl)								
Cell	X1	X6	X11	SRY	ND3	Nanodrop (ng/μl)	Specific genomic (%)	Specific mitochondrial (%)	Fold-amplification (copies)

1	133	22.8	2.36	78.0	81.8	112	52.7	73	1.1×10^6^
2	27.1	10.7	7.1	41.6	293	51	42.4	574	4.3×10^5^
3	25.6	33.8	4.5	48.1	193	58	48.2	332	5.5×10^5^
4	21.8	23.8	17.9	75.1	151	77	45.0	196	6.9×10^5^
5	25.9	40.4	2.3	67.5	266	56	60.7	475	6.7×10^5^
6	22.2	6.14	13.9	36.7	55.5	46	42.8	120	3.9×10^5^
7	30.4	67.5	1.8	153	1006	144	43.8	698	1.2×10^6^
8	8.06	12.6	2.7	13.5	882	50	18.4	1764	3.8×10^5^

Cells 1–8 (mean ± std)							44.2 ± 12	529 ± 545	6.9×10^5 ^± 3.4×10^5^

These results indicate that WGA products from single cells consisted of ~72% non-specific DNA and ~28% specific DNA, and that the specific DNA contained ~700000 copies of each successfully-amplified locus.

### P53 gene locus

A 240 bp fragment (P53short), containing exon 8 of the mouse P53 gene was successfully amplified from 17/20 cells from fresh tissue sections, and from 5/17 cells from pre-stored tissue sections. Amplified fragments were purified and sequenced (Figure 2C), and mutations were detected in some samples (see below). In order to see whether a longer fragment of the same locus could also be amplified in cells that showed successful amplification of P53short, a second PCR was performed, with primers for P53long, a 2.5 kbp fragment encompassing the P53short fragment. 12/22 cells showed successful amplification of the P53long fragment. Of these, 9 were from fresh sections and 3 were from pre-stored sections.

### Mitochondrial DNA

For each cell, we amplified by PCR and analyzed by gel electrophoresis the ND3 mitochondrial locus. Amplification was successful for all 20 cells from fresh tissue sections, and for 13/17 cells from pre-stored tissue sections (Figure 2D). The ND3 locus was also amplified in 8 cells by quantitative real-time PCR. ND3 was amplified with greater efficiency than genomic loci, achieving a mean specific yield of 529% (Table 4). However, in contrast to the hemizygous genomic loci, which were known to be present at a single copy in the template DNA, the copy number of mitochondrial genomes was not known in either the single cell samples or in the tail clipping DNA that was used for the dilution series. Therefore, it was not possible to calculate precise fold – amplification for mitochondrial DNA.

### Mutations

In this study, the mouse used for experimentation was a mismatch-repair deficient mouse, with a knockout of the MLH1 gene (see materials and methods). These mice are known to accumulate somatic mutations in MS loci in an accelerated rate [47]. In order to analyze somatic mutations, for each cell the length of amplified MS loci was compared to the length of the corresponding MS loci amplified from DNA obtained from the tail clipping of the same animal (obtained by standard procedures and without WGA). Replication slippage mutations (insertions or deletions of basic repeat units) were detected as differences in the size of the fragments (Figure 2B). A total of 994 slippage mutations were detected, representing 39.6% of all amplified alleles. Quantitative analysis of these mutations was performed allowing for reconstruction of the cells' lineage tree (Frumkin, D. *et al*., submitted), and for estimation of the depth of the cells (Wasserstrom, A. *et al*., submitted). Analysis of the genomic sequences from exon 8 of the P53 gene revealed no mutations in the normal lung epithelium cells, whereas 9 tumor cells were found to have the same specific point mutation, which is known to be associated with cancer (Frumkin, D. *et al*., submitted). In several tumor cells, sequencing revealed the presence of both the normal and mutated alleles (Figure 2C), indicating that the cells were heterozygote at the P53 locus.

### Contamination

All single cell samples that were catapulted from a particular tissue section were subsequently accompanied in all procedures by a negative control sample that consisted of a 100 μm^2 ^piece of empty membrane catapulted from the same tissue section. A total of 8 negative control samples (from 8 different tissue sections) were produced, and each was amplified by 58 PCRs (24 multiplex + 34 singleplex). A positive signal was detected in 8 out of the 564 PCRs that were performed on negative control samples. Upon detection of each positive signal in a negative control sample, the primer mix was replaced by a fresh mix prepared from stock solutions, and in all cases subsequent PCRs of negative control samples with the fresh mixes did not produce any signal. These results indicate that negative control samples were not contaminated with DNA, and that the observed signals in PCRs of negative control samples were a result of contamination of the primer mixes during liquid handling.

## Discussion and Conclusion

The amplification procedure described here can be optimized further, to allow for more efficient cell capture, higher amplification rates, and lower ADO rates. However, even with best optimization, it is unlikely that laser micro-dissected cells from tissue sections could achieve the same amplification rates as fresh cells from cell suspensions. Truncation of some cells during sectioning, and direct damage to DNA from the laser beam represent intrinsic limitations of the method that are not likely to be overcome. However, lower levels of amplification are not necessarily expected to pose a serious problem for potential applications that are centered on large scale genomic surveys.

### Cell capture

Laser catapulting into adhesive caps was relatively inefficient as 60% of catapulting attempts resulted in cells landing back onto the slide surface at a nearby location, rather than sticking to the caps. This did not pose a significant problem for us because the processes of laser micro-dissection, catapulting, and detection of failed catapulting attempts were very fast compared to upstream and downstream procedures. Typically, cells were micro-dissected and catapulted within less than a minute and visual scanning of slides after catapulting enabled the detection of failed attempts within seconds. This amount of time was insignificant compared to the time spent on tissue sectioning and staining, WGA, PCR, capillary electrophoresis, and signal analysis. Initially, we spent a considerably longer time visually scanning caps after catapulting in order to verify the presence of cells. This procedure, however, was discontinued when it became apparent that it was not necessary. Out of 41 cells that were catapulted and that "passed" visual inspection of the slides, only 4 (9.7%) failed to amplify in subsequent WGA, indicating that visual scanning of slides was sufficient in order to detect > 90% of catapulting failures. In addition, the cells that were analyzed came from large, homogenous populations, and therefore cells that failed to catapult could easily be substituted in the experiment with nearby similar cells. Therefore, low catapulting efficiency did not represent a bottleneck in our application and we did not attempt to optimize this process. However, in applications where the desired cells are part of rare populations (e.g. micro-metastatic cells), attempting to increase catapulting efficiency may be beneficial. One possibility for achieving this could be by minimizing the distance from the cap to the tissue section, as capturing is more efficient across small distances. This step, however, carries the risk of contamination from unnoticed contact between the cap and tissue. A second option would be to use non-adhesive caps filled by lysis buffer, instead of adhesive caps. Liquid may be more efficient than solid adhesive material in capturing catapulted cells, but this approach would require manual filling of each individual cap immediately prior to catapulting if evaporation of the small volume of liquid is to be avoided. Alternatively, catapulting energy and focus could be changed from default values and optimized for best efficiency. Increased energy level is expected to provide more kinetic energy to catapulted cells, but it also carries the risk of causing damage to DNA. By performing such optimization steps and with experienced handling, it should be possible, according to the manufacturer of the laser machine, to achieve catapulting efficiency of over 90%.

### Effects of pre-storage and multiplexing on amplification rates

Amplification success rates were significantly higher in cells obtained from freshly prepared tissue sections compared to cells obtained from sections that were mounted and stored at -80°C for several weeks prior to WGA. In the absence of nucleases, DNA is expected to be stable for years, and therefore the reduction in amplification rates was most probably caused indirectly, by biochemical processes in the tissues during storage. DNA degradation in immunohistochemically stained slides was previously reported to occur via oxygen radicals as a result of exposure to air [33]. Another possibility might be increased nuclease activity during storage due to temperature changes or other unidentified technical problems. Amplification rates in singleplex PCRs were significantly higher in comparison to multiplex PCRs for both fresh and pre-stored samples. Poorer performance of multiplex PCR on single cell WGA products was reported previously [26], and likely results from preferential amplification of loci in multiplex PCR compounded by differing copy numbers of different genomic targets in WGA products. Preferential amplification often occurs in multiplex PCR as a result of different efficiencies of different primer pairs and from creation of primer dimers, which are more likely to occur as the number of primers increases [48]. This problem is compounded when the template DNA contains variable copy numbers at different loci. Although multiple displacement amplification is the method that results in the most balanced genome amplification to date, it still suffers from up to 6 fold amplification representation differences between loci [49]. Therefore, in multiplex PCR on multiple displacement amplification products, when primer pairs with relatively low efficiency act on genomic loci with relatively low copy numbers, the result might be amplification failure or ADO. This problem could be circumnavigated by optimization of the multiplex PCR, by changing primer sequences, concentrations, and cycling conditions. However, such optimization might prove more labor intensive and time consuming than performing singlplex PCRs. In this study, the amount of WGA product from each cell was sufficient for 120 PCRs. Furthermore, as the volume of the multiple displacement amplification reaction can be scaled up, there is no theoretical limit to the amount of PCRs that could be performed on each cell.

### Comparison of amplification rates with other studies

Comparison of the efficiency of the single cell WGA presented here with results from previous studies is hampered by the lack of a universally accepted nomenclature. The term ADO was defined here and elsewhere [26,33,50] as failure of amplification of one out of two alleles in a heterozygous locus. By this definition, failure of amplification of both alleles in a heterozygous locus does not count as ADO, but rather counts as "amplification failure". However, other studies [30,51] have defined ADO as the failure of amplification of any of the 2 alleles in a heterozygous locus, and by this definition failure of amplification in 2 alleles is counted as 2 ADO events, and the term "amplification failure" is not used. Yet other studies used the term ADO without providing any definition, making it difficult to interpret their results. Similar ambiguity exists in other commonly used terms, such as "amplification efficiency", "genomic coverage", and "allele calling", therefore necessitating careful scrutiny and adjustment of values when comparing results from different studies.

WGA was previously performed on many types of single cells from cell suspensions, with varying success rates, depending on the specific method used and on the specific cell type that was analyzed. Of all WGA methods, multiple displacement amplification gives the most complete genomic coverage [6], and is considered the most effective method to date [7]. Multiple displacement amplification was recently demonstrated on single buccal cells, achieving 90% Amp/L and 72% Amp/A [30]; on single blastomeres, achieving 90.3% Amp/L, 67.5% amp/A, and 25.5% ADO [26]; and on single lymphoblasts, achieving 89.5% Amp/L, 78% Amp/A, and 25.8% ADO [29,52]. These amplification rates are higher than the corresponding rates presented here (70.3% Amp/L, 63.5% Amp/A, 64.2% ADO from fresh tissue sections).

To the best of our knowledge, multiple displacement amplification was not previously performed on single cells from frozen tissue sections, and consequently it is currently unknown whether fresh suspended cells are generally expected to amplify better than cells from frozen tissue sections. However, a handful of studies have performed PCR directly (without WGA) on single cells from frozen tissue sections, achieving rates of amplification that are similar to or slightly lower than the rates presented here. In one study, manually micro-manipulated single cells from a human skin section were amplified with 37% Amp/A [2]. In another study, Laser micro-dissected single keratinocytes were amplified with 70% Amp/L, 52.5% Amp/A, and 50% ADO [53].

Unlike formalin fixation, which causes degradation [54] and cross-linking [33] of DNA, and thereby resulting in poor amplification from single cells [33], freezing of tissues is not expected to significantly damage DNA. However, tissue sectioning might cause truncation of nuclei, and it has been estimated that when processing single cells from 6 μm sections, truncation leads to a loss of 10–20% of the genetic material [33]. In this study, cells with an average nucleus diameter of ~5 μm were isolated from 6 μm sections, and since cells are not precisely aligned on the horizontal plain, it is indeed likely that truncation of nuclei contributed to loss of genetic information. This possibility gains strength from the observation that the concordance of amplification status in loci that were located on the same chromosome was significantly higher than the concordance of amplification status in loci that were located in different chromosomes, and from the negative correlation that was observed between physical distances between loci on the same chromosome and amplification concordance. The problem of genetic loss due to nuclei sectioning could be overcome by using thicker sections, but this might result in some samples containing more than a single nucleus. In addition, the use of thicker sections requires higher laser energy levels for micro-dissection, and since laser energy might damage DNA (see below), it would require the use of larger "safety" margins around micro-dissected nuclei.

The use of laser energy for micro-dissection might also contribute to loss of genetic material by causing direct damage to DNA. According to the manufacturer of the laser machine, such damage is expected to be confined to an area immediately adjacent to the ablation path, with the width of the damaged area not more than 50% of the ablation path width (e.g. a damaged strip with a width of 0.5 μm on either side of a 1 μm wide ablation path). For this reason, ablation path widths should be minimized by using the lowest energy levels possible for micro-dissection.

Considering this, it is likely that WGA of laser micro-dissected cells from tissue sections can achieve somewhat lower amplification rates than WGA of fresh cells from suspension. Despite this, it should be noted that lower rates of amplification are not expected to pose a significant problem in most future applications of the work presented here. Until the present, most single cell WGA studies have been motivated by applications in pre-implantation genetic diagnosis, and since ADO represents the greatest challenge in this field (potentially resulting in misdiagnosis of embryos), much emphasis was put on reducing ADO rates to the minimum. However, the potential applications of single cell WGA from tissue sections are not in pre-implantation genetic diagnosis, but rather in research areas where large scale genomic surveys are employed at the single cell level, such as in the emerging fields of cell lineage [14,55,56], reviewed in [57], cell depth [56], (Wasserstrom, A. *et al*, submitted), and stem cell dynamics [58] analyses. These fields are not focused on comparing genotypes of cells in any particular locus, but rather extract information from a general genetic comparison performed across multiple loci. In these types of researches, the rate of ADO has little significance, and the most important parameter may be the total amount of genetic information that can be obtained from each cell in a given amount of time, labor, or money.

### Possible applications

As stated above, genome amplification of single cells from laser micro-dissected tissues can be very beneficial for cell lineage and depth analyses. Indeed, the motivation for the work presented here came from our wish to expand the capabilities of the method for cell lineage analysis that was recently developed by our group [55]. Previously, in-vivo cell lineage reconstruction and depth estimation was performed on non-adherent cells or on cells isolated by enzymatic digestion of tissues [14], (Wasserstrom, A. *et al*, submitted). With the development of the protocols presented here, it became possible to use laser micro-dissection for analyzing lineage relations between specific cells in tissue micro-environments. We used mutational information from the genomes of the 37 micro-dissected cells described here to reconstruct their lineage tree. Analysis of this reconstructed tree revealed that the cancer cells shared a common clonal origin and that they were significantly deeper (i.e. had undergone more cell divisions) than adjacent normal lung epithelial cells. It also revealed that growth of the tumor occurred in a coherent manner, without significant cell motility (Frumkin, D. *et al*., submitted).

Correlating between a single cell's genome and its' morphology and precise position in the tissue micro-environment may also open new research possibilities in the study of the genetics of various physiological and pathological processes. For example, in the physiological context, it could be used to determine whether genetic alterations underlie phenotypic changes associated with aging, or to determine the exact spatial and temporal pattern of generation of receptors in immune cells and meiotic recombination in germ cell precursors. It could also be used to study genetic changes underlying many pathological processes, and especially cancer. For example, this work may advance understanding of the initiation and progression of pre-malignant and cancerous lesions, by allowing for comprehensive correlations to be made between the phenotype of various abnormal cells and possible mutations in various proto-oncogenes.

Tumor tissues are composed of heterogeneous cell populations. A minority of tumor cells are "cancer stem cells" that may be important for resistance to therapy and metastasis [59]. The tumor microenvironment contains various non-malignant cells such as lymphocytes and fibroblasts that interact with the malignant cells [60]. The ability to analyze, at the single cell level, the genomes of various malignant and non-malignant cells is expected to increase our understanding of cancer.

The amplification rate of the mitochondrial ND3 locus was very high in this study, as all 20 cells from fresh sections showed amplification. This result is not surprising considering the high copy number of mitochondrial genomes relative to the single copy of hemizygous genomic loci in a single cell. In addition, the ND3 locus achieved a high specific yield of > 500% in the quantitative real-time PCR, possibly as a result of the high efficiency of the multiple displacement amplification on circular DNA templates, relative to linear fragments. This result indicates that the method may allow for efficient screening of cells for the presence of other circular DNA species, including genomes of intracellular pathogens. Such a screening may be used to investigate the precise pattern of the spread of pathogens and to uncover cell populations that harbor latent viral infections.

## Methods

### Experimental animals

Mlh1+/- mice were obtained from Michael Liskay (OHSU, described in [47]) and were maintained at our institute under a dual genetic background (C57Bl/6 and 129SvEv). All work was done under the Weizmann Institute of Science IACUC approval. Mlh1+/- C57Bl/6 and Mlh1+/- 129SvEv were mated to yield an Mlh1-/- animal that was used for the experiment. At the age of 9 months, upon signs of illness, the animal was sacrificed by CO_2 _asphyxiation. Examination revealed a round tumor mass (diameter – 9 mm) in the thoracic cavity and two additional tumor foci in the right and left lungs. Tissues from the large tumor mass and from both lungs were removed, snap frozen in liquid nitrogen, and stored in -80°C until use.

### Preparation of tissue sections

Frozen mouse tissues were cut in a cryostat microtome (CRYOTOME – LEICA CM3050 S) at -20°C to 6 μm sections and mounted on membrane-coated slides (PALM MembraneSlides – 1 mm PEN membrane covered). Tissue sections were stained with Hematoxylin and Eosin solutions (Sigma-Aldrich) according to the following protocol (all solutions were ice-cold): 2 minutes in 70% ethanol followed by several rinses in double-distilled water (DDW), 2 minutes in Hematoxylin, 2 minutes in tap water pre-filtered with 0.2 μm disposable filter units (Schleicher & Schuell), several brief rinses in Eosin, several rinses in 70% ethanol, several rinses in 100% ethanol. Following staining, tissue sections were dried at room temperature for 5 minutes prior to laser micro-dissection.

### Laser assisted micro-dissection

Laser micro-dissection was performed using the PALM MicroBeam micro-dissection apparatus (PALM Microlaser Technologies). Parameters for laser energy, focus, and speed were adjusted individually for every tissue section, such that dissection was performed with minimal laser energy. The minimal energy level was determined by performing continuous laser micro-dissection with decreasing energy levels on a portion of the section adjacent to the area destined for cell isolation. Single cell samples were catapulted using default catapulting energy and focus parameters into adhesive caps of 200 μl micro-tubes (PALM Microlaser Technologies). In ~60% of catapulting attempts the catapulted cells failed to adhere to the adhesive caps of the micro-centrifuge tubes, and instead were observed to land back onto the tissue sections at nearby locations. In these cases, the micro-tubes were discarded and the micro-dissected cells were not processed further. In 41 cases (representing ~40% of attempts) catapulting appeared to be successful, and for these a visual inspection of the caps was performed. Cells that adhered to the central, relatively flat area of the caps were detected, but cells that adhered to the peripheral concave areas of the cap could not always be identified with certainty. In order to verify successful catapulting, a preliminary PCR assay was performed on all 41 samples following DNA extraction and WGA.

### DNA extraction

DNA extraction from single cells was based on the protocol of the GenomiPhi DNA amplification kit (Amersham Biosciences) for extraction of DNA from blood cells, with modifications. For each sample, 4 μl lysis buffer (composed of 50% PBS and 50% of the following solution: 400 mM KOH, 100 mM DTT, 10 mM EDTA, 3% Tween-20) was applied directly to the adhesive cap containing the catapulted cell sample. The tube was then closed and the sample was placed on ice for 15 minutes. After lysis, neutralization was performed by adding 2 μl neutralization buffer (400 mM HCL, 600 mM Tris HCL, PH 0.6). DNA extraction from the mouse tail clipping was performed with the Wizard SV Genomic DNA purification system (Promega).

### Whole genome amplification

WGA was performed in the original micro-centrifuge tube containing extracted DNA using the GenomiPhi DNA amplification kit (Amersham Biosciences) according to the manufacturer's protocol, with all reaction volumes increased six-fold (final volume – 120 μl).

### Preliminary PCR assay

A preliminary multiplex PCR was performed (with primers for D6MIT36, D10MIT233, D1MIT132, M2 – see Table 1) for each WGA product. Reagents and thermal cycling conditions were the same as for the other PCR reactions (see below). Out of 41 samples, 4 showed no amplification in any locus and were thus considered to contain no cell and were discarded. The remaining 37 samples were processed further by multiple PCRs followed by capillary electrophoresis and signal analysis.

### Contamination control

Prior to work, gloves and work surfaces were pre-rinsed with DNA away solution (Molecular BioProducts). The microscope plate was cleaned with Iso-propanol and the cryotome surfaces were cleaned with acetone. Cutting of tissue sections was performed using a new blade for each section. For staining, a new set of solutions was prepared and used for each section.

Negative control samples for WGA were prepared for each mounted and stained slide from a 100 μm^2 ^piece of empty polyethylene membrane containing no mouse tissue. DNA extraction and WGA were performed in a protected workstation (Cleanspot PCR workstation – COY laboratory products) that was used solely for these procedures, with a set of pipettes that were used solely for these procedures and that were kept inside the workstation at all times. Prior to DNA extraction and WGA, the contents of the workstation (including pipettes) were exposed to UV light for one hour.

### Primer design, PCR amplification, and electrophoresis

A total of 129 genomic loci and the ND3 mitochondrial locus were amplified for each single cell sample and for the tail clipping DNA. Five loci that failed to amplify in tail clipping DNA were not analyzed further. The remaining 124 loci are listed in Table 1. Loci 1–90 were amplified in multiplex reactions, each containing 3–4 primer pairs, and loci 91–124 were amplified in singleplex reactions. Loci 1–56 were amplified using commercial primers (ABI PRISM^® ^Mouse Mapping Primers v.1.0.) and therefore the sequences for these loci are not available. Sequences of primers for locus 121 (SRY) were obtained from [61], and sequences of primers for locus 124 (ND3) were obtained from [62]. Sequences of primers for all other loci were designed using Primer3 [63] with the following parameters changed from default: Primer size = 20,22,27 (minimum, optimal, maximum); Primer Tm = 62°C, 65°C, 68°C (minimum, optimal, maximum); Max Tm difference = 2.5°C; CG clamp = 1. All PCR reactions were carried out in a volume of 25 μl including 1.2 μl unpurified WGA product as template, 0.2 μM of each primer, 0.2 mM of each dNTP (BIOLINE), 0.625U of Thermo-Start DNA Polymerase (ABgene), and 2.5 μl of 10× PCR buffer (ABgene). Thermal cycling conditions for all reactions (except for the long range PCR reaction) were : (i) 15 minutes 95°C, (ii) 35 cycles of: 1 minute 95°C, 1 minute 58°C, 1 minute 72°C, (iii) 15 minutes 72°C.

Thermal cycling conditions for the long range PCR reaction (with P53long primers) were: (i) 15 minutes 95°C, (ii) 35 cycles of: 30 seconds 95°C, 30 seconds 58°C, 3 minutes 72°C, (iii) 15 minutes 72°C.

Most amplified products were run on a capillary electrophoresis machine: 0.75-1 μl of a PCR product was added to a 14 μl solution containing 1 part GeneScan 500 LIZ size standard (Applied Biosystems) and 24 parts HiDi Formamide (Applied Biosystems), mixed thoroughly, and run on an ABI prism 3130xl Genetic Analyzer machine (Applied Biosystems). Fragment analysis was performed using the GeneMapper v3.7 software accompanying the machine.

Amplified products of P53short, P53long, ND3, and SRY loci were run on a gel: 5 μl of each PCR product was mixed with 3 μl of Blue/Orange 6X loading dye (Promega), loaded to a well of a gel containing 0.8–1.2% agarose (Agarose Low EEO – Hispanagar, Spain), and stained with 50 μg Ethidium Bromide (amresco). DNA ladders used were 50 bp DNA step ladder (Promega) and 1 KB DNA ladder (NEB). Products were subject to electrophoresis at 100 V for 1 hour and gels were photographed with ImageMaster (Pharmacia Biotech).

### Robotic automation

We used a programmable laboratory robot (TECAN Genesis) augmented with a PCR machine (Biometra TRobot) to perform the liquid handling for PCR, the PCR itself, and the sample preparation for the capillary and gel electrophoresis.

### DNA yield and size range

Prior to calculation of yield, WGA products were purified by alcohol precipitation, according to the protocol provided in the manual of the GenomiPhi DNA Amplification Kit (Amersham Biosciences). DNA concentrations of purified WGA products (in ng/μl) were measured with the Nanodrop ND-1000 Spectrophotometer (Nanodrop technologies), and results were multiplied by 120 μl (total volume of each WGA reaction), yielding total DNA yields. WGA products of cells 1–8 were analyzed for size-range of products by gel electrophoresis using the reagents and conditions that are outlined above (in "Primer design, PCR amplification, and electrophoresis").

### Quantitative real-time PCR

Quantitative real-time PCR was performed with the MyiQ single color Real-Time PCR Detection System (Bio-Rad). Each reaction contained 1 μl of WGA product as template, 0.2 μM of each forward and reverse primer, 12.5 μl SYBR Green Supermix (Bio-Rad), and DDW to a total volume of 25 μl. Serial dilutions of tail-clipping DNA were used for the dilution series. Thermal cycling conditions for all reactions were 10 minutes 95°C followed by 45 cycles of: 1 minute 95°C, 1 minute 58°C, 1 minute 72°C.

Percentage of specific genomic DNA for each cell was calculated by dividing the mean specific DNA concentration for genomic loci (X1, X6, X11, and SRY) as measured by real-time PCR, by the total (specific + non-specific) DNA concentration, as measured in the Nanodrop spectrophotometer. Percentage of specific mitochondrial DNA was calculated similarly from the results for the ND3 locus. Fold-amplification was calculated for genomic loci only by multiplying the specific genomic DNA percentage by the total DNA yield, and dividing the result by the approximate weight of a single mouse diploid genome (6 pg).

### Statistical analysis

P values for all comparisons of Amp/L, Amp/A, and ADO rates between multiplex vs. singleplex loci and between fresh vs. pre-stored tissue sections were calculated using the Kolmogorov-Smirnov goodness-of-fit hypothesis test as implemented by MATLAB (MathWorks, Natick, Massachusetts, United States). P value for the comparison between ADO rates of long vs. short alleles was calculated using the binomial distribution with parameter 0.5.

## Authors' contributions

DF performed the experiments and drafted the manuscript. AW, SI, and AH performed the experiments. GR and ES coordinated the experiments and helped to draft the manuscripts. All authors read and approved the final manuscript.

## Supplementary Material

Additional File 1 Gel electrophoresis of whole genome amplification products. Click here for file

Additional File 2 Amplification and dropout rates for cells. Click here for file
