# Supplementary material for: Amplification of multiple genomic loci from single cells isolated by laser micro-dissection of tissues
Source: BMC Biotechnol. 2008 Feb 20;8:17. doi: 10.1186/1472-6750-8-17 (PMC2266725; doi:10.1186/1472-6750-8-17)
Supplement: Additional File 1 — Gel electrophoresis of whole genome amplification products. [file 1472-6750-8-17-S1.doc]

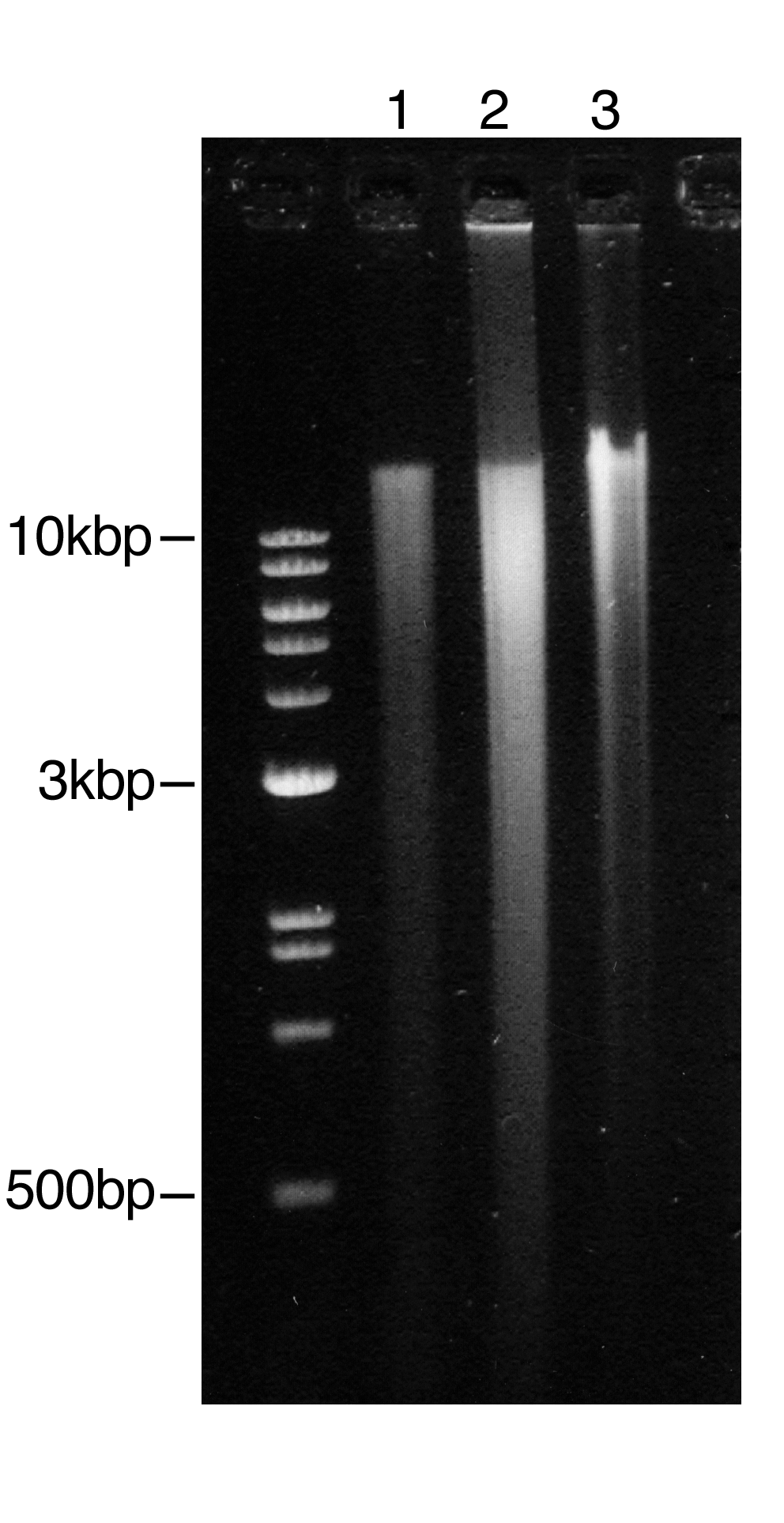


**Additional File 1.** Gel electrophoresis of WGA products. Lane 1 – genomic DNA extracted from a tail clipping, without WGA. Lane 2 – WGA product of DNA extracted from a single cell. Lane 3 – WGA product of a negative control sample (no DNA template).
