# Supplementary material for: Amplification of multiple genomic loci from single cells isolated by laser micro-dissection of tissues
Source: BMC Biotechnol. 2008 Feb 20;8:17. doi: 10.1186/1472-6750-8-17 (PMC2266725; doi:10.1186/1472-6750-8-17)
Supplement: Additional File 2 — Amplification and dropout rates for cells. [file 1472-6750-8-17-S2.doc]

**Additional File 2.** Amplification and dropout rates for cells

|  | **Singleplex PCR** | | | **Multiplex PCR** | | |
| --- | --- | --- | --- | --- | --- | --- |
| Cells from fresh tissue sections (1-20) | | | | | | |
| cell | Amp/L  (%) | Amp/A  (%) | ADO  (%) | Amp/L  (%) | Amp/A  (%) | ADO  (%) |
| 1 | 68.7 | 62.1 | 66.7 | 83.1 | 56.7 | 71.2 |
| 2 | 68.7 | 59.4 | 66.7 | 74.1 | 50.0 | 70.9 |
| 3 | 81.2 | 70.2 | 83.3 | 73.0 | 46.3 | 81.0 |
| 4 | 84.3 | 81.0 | 33.3 | 86.5 | 59.7 | 67.6 |
| 5 | 75.0 | 70.2 | 50.0 | 73.0 | 54.2 | 60.6 |
| 6 | 62.5 | 62.1 | 50.0 | 65.1 | 46.3 | 66.0 |
| 7 | 68.7 | 59.4 | 83.3 | 65.1 | 47.5 | 59.1 |
| 8 | 65.6 | 59.4 | 83.3 | 52.8 | 30.4 | 92.8 |
| 9 | 65.6 | 56.7 | 80.0 | 66.2 | 46.9 | 64.7 |
| 10 | 62.5 | 54.0 | 80.0 | 66.2 | 46.3 | 66.6 |
| 11 | 93.7 | 83.7 | 66.7 | 71.2 | 46.2 | 78.1 |
| 12 | 59.3 | 54.0 | 66.7 | 75.8 | 49.3 | 76.7 |
| 13 | 96.8 | 91.8 | 33.3 | 78.1 | 59.3 | 54.2 |
| 14 | 46.8 | 37.8 | 100 | 57.7 | 34.9 | 86.6 |
| 15 | 59.3 | 51.3 | 100 | 47.7 | 30.1 | 80.5 |
| 16 | 93.7 | 91.8 | 16.7 | 78.8 | 48.8 | 83.6 |
| 17 | 84.3 | 78.3 | 50.0 | 74.4 | 48.8 | 75.8 |
| 18 | 37.5 | 29.7 | 100 | 34.4 | 21.0 | 84.6 |
| 19 | 56.2 | 48.6 | 75.0 | 51.1 | 31.3 | 84.6 |
| 20 | 75.0 | 67.5 | 0 | 63.3 | 53.6 | 31.9 |
| Cells from pre-stored tissue sections (21-37) | | | | | | |
| 21 | 34.3 | 32.4 | 66.6 | 45.4 | 27.1 | 87.8 |
| 22 | 31.2 | 24.3 | 100 | 29.5 | 17.9 | 86.9 |
| 23 | 18.7 | 18.9 | 66.6 | 25.0 | 16.0 | 80.0 |
| 24 | 56.2 | 48.6 | 75.0 | 21.5 | 12.9 | 86.6 |
| 25 | 53.1 | 48.6 | 75.0 | 37.5 | 22.2 | 88.4 |
| 26 | 50.0 | 43.2 | 100 | 51.1 | 30.8 | 86.8 |
| 27 | 21.8 | 16.2 | 100 | 54.4 | 32.5 | 88.3 |
| 28 | 56.2 | 45.9 | 100 | 55.5 | 33.1 | 88.1 |
| 29 | 21.8 | 21.6 | 0 | 22.2 | 13.8 | 84.2 |
| 30 | 37.5 | 35.1 | 75.0 | 42.2 | 26.5 | 81.8 |
| 31 | 18.7 | 18.9 | 66.6 | 52.2 | 33.1 | 80.0 |
| 32 | 21.8 | 18.9 | 100 | 40.9 | 25.9 | 81.2 |
| 33 | 59.3 | 59.4 | 25.0 | 46.5 | 27.1 | 91.6 |
| 34 | 25.0 | 21.6 | 100 | 59.0 | 35.8 | 86.6 |
| 35 | 31.2 | 29.7 | 75.0 | 30.6 | 19.7 | 78.2 |
| 36 | 59.3 | 54.0 | 60.0 | 48.8 | 30.2 | 84.2 |
| 37 | 25.0 | 21.6 | 100 | 25.0 | 14.8 | 90.0 |
